# Feature Papers in Bone Biomaterials

**DOI:** 10.3390/jfb15120365

**Published:** 2024-12-03

**Authors:** Zifei Zhou, Feng Chen

**Affiliations:** 1 Department of Orthopedics, Shanghai Tenth People’s Hospital, School of Medicine, Tongji University, Shanghai 200072, China; 2 Shanghai Key Laboratory of Craniomaxillofacial Development and Diseases, Shanghai Stomatological Hospital & School of Stomatology, Fudan University, Shanghai 201102, China

## 1. Introduction

Bone biomaterials have garnered significant attention in the field of regenerative medicine due to their application in a wide range of clinical scenarios. These materials are often designed to mimic the properties or contents of natural bone. The primary focus for researchers lies in enhancing their biocompatibility, mechanical strength, and osteoconductivity, essential attributes for successful bone integration and healing [1]. The biomaterials used for bone regeneration can be categorized into several different types, such as metal, ceramic, polymer, and composite biomaterials. Advancements in the structure of biomaterials have also further expanded their potential. Nanostructured materials exhibit unique properties that enhance cellular responses and promote bone regeneration at the molecular level. This Special Issue contains 10 papers: 8 articles which explore several newly designed bone biomaterials and 2 reviews which explain the importance of calcium phosphate and LIPUS stimulation.

## 2. Overview of Published Articles

The use of photobiomodulation as a biological intervention therapy is promising, as it may improve bone matrix protein deposition, periosteum development, and trabecular matrix production. It has been found that photobiomodulation does not increase the formation of new bone in critical-size defects when combined with deproteinized bovine bone mineral and collagen membrane. However, absorption of deproteinized bovine bone mineral could be accelerated using photobiomodulation; in such cases, new bone formation has been observed around the collagen membrane, surpassing the original boundaries of the defect [2].

Functional pressure from muscles and ligaments near the hyoid can cause biological apatite crystallites, periosteal insertions, and fibrocartilage entheses to adopt a preferred orientation, in addition to increasing bone mineral density. This indicates that functional pressure at these locations could have a significant impact on the morphological characteristics of the entheses, as well as in bone quality [3].

It has been widely proven that both bone marrow mesenchymal stem cells (BMSCs) and adipose-derived stem cells (ASCs) show great potential in bone repair. Furthermore, the recapitulated osteogenesis ability of BMSC or ASC spheroids makes them an advanced tool in bone regeneration. ASC spheroids seeded in a 3D-printed scaffold showed a good spreading morphology and secreted vascular endothelial growth factor. In 3D-printed scaffolds with ASC spheroids attached, new bone tissue formation was demonstrated through histological in vivo results [4].

Biodegradable metallic biomaterials (BMBs) are promising candidates for orthopedic implants. Among the BMBs available, Mg and its alloys demonstrate multiple biological effects, including osteogenic and angiogenic properties. Compared to pure Mg, the Mg-Sc-Sr alloy induced a higher osteoblastic differentiation of BMSCs and angiogenic differentiation of HUVECs. The Mg-Sc-Sr alloy is an attractive candidate for future bone implants [5].

Various calcium phosphate (CaP)-based biomaterials have been used in bone repair. Hou et al. reviewed the influence of CaPs’ species, sizes, and morphologies on its physicochemical properties and biological behavior during the promotion of bone regeneration. This review will be beneficial in understanding the characteristics of CaPs and in creating new high-performance biomaterials for bone repair [6].

Biophysical stimulations play a role as non-invasive and controllable therapy in clinical practice. Moreover, the application of biophysical stimulations could work as “biophysical switch” in the treatment of diseases, with or without biomaterials. Among these biophysical stimulations, low-intensity pulsed ultrasound (LIPUS) has shown many advantages when combined with musculoskeletal-related biomaterials. Jia et al. present a review regarding the synergistic action of LIPUS and biomaterials in the repair of bone, cartilage, or nerves and in the treatment of osteonecrosis and osteolysis. Their crucial acoustic excitation parameters and current limitations and future perspectives are discussed [7].

Wood-derived materials are another candidate for bone scaffolds, as they exhibit similar density and mechanical properties to those of bone tissue. Andze et al. produced a dry-state wood material using a three-step method, which included partial delignification, extraction, and densification. The swelling property of this wooden material was reduced after a chemical treatment. Moreover, the wood sample showed good cytocompatibility [8].

Immunoregulatory topography could modulate the body’s response to the surface of biomaterials. RAW264.7 macrophages showed different responses to nanopillar and nanopit substrates. The morphology of the macrophages was large and spread well due to the topography of the nanopillars, which had a diameter of 450 nanometers and an inter-pillar space of 300 nanometers. A significant increase in cell elongation was observed in the macrophages in response to nanopits with a depth of 150 nm and 800 nm edge–edge spaces. The phenotypic markers and integrin β1 expression of M2 macrophages were elevated and inflammatory cytokines we less expressed after these cells exhibited spreading or elongation [9].

Bone tissue engineering can greatly benefit from three-dimensional printing technology. Such technology can enable users to craft personalized designs, create prototypes rapidly, and fabricate intricate scaffolds [10]. Varisized scaffolds were fabricated using three-dimensional printed scaffolds formed of polylactic acid (PLA) and different amounts of gelatin and chitosan (CH) hydrogel. The behaviors of human bone mesenchymal stromal cells (hBMSCs) were unaffected by the hydrogel’s content or the core–shell structure’s geometry. All scaffolds have the potential to promote cell adhesion, proliferation, and osteogenic differentiation. Future studies could be aided by the strut thickness, hole height, hole width, and CH contents of the PLA-CH three-dimensional scaffolds described in this article [11].

Mn can be introduced onto the surface of titanium platforms by employing the plasma immersion ion implantation and deposition technique, leading to the surface having heightened zeta potentials and nano-hardness. Under mono-culture conditions, a Mn scaffold could stimulate the M1 phenotypes of Raw264.7 macrophages while displaying little promotion of cellular osteogenic differentiation in mouse BMSCs. However, a significant transformation to the M2 phenotypes of these macrophages and significant osteogenic differentiation of the mouse BMSCs were observed when they were co-cultured together. Consequently, Mn-modified biomaterials demonstrated the ability to trigger immunomodulation and stimulate osteogenic differentiation [12].

## 3. Conclusions and Future Perspectives

Bone biomaterials are an interdisciplinary field within bone tissue engineering [13]. Continuous basic and preclinical research is required to address the difficulties in bone repair. A thorough understanding of the interaction mechanisms between bone biomaterials and bone tissue is essential for the development of personalized bone biomaterials. We should encourage the study of the interaction of bone biomaterials with bone and its surrounding tissues, and especially the influence multiple cells in the local microenvironment have on bone biomaterials. Advances in these areas will enable the design and manufacture of bionic bone biomaterials that are more suitable for bone repair processes.

When designing bone biomaterials, the balance between the degradation of biomaterials and the bone regeneration process is another factor to be considered. If the degradation rate of the biomaterial is consistent with the regeneration process of the bone tissue, then it can be ensured that the biomaterial scaffold will support the formation of new bone tissue. Achieving this optimal balance prevents the premature loss of the structure of the biomaterial while it is still providing continuous mechanical support to bone tissue.

Multiple physical stimuli, such as near-infrared, LIPUS, or electrical signals, when combined with biomaterials, can also further accelerate the bone regeneration process. The combination of physical stimulation and biomaterials often promotes cell proliferation, osteogenic differentiation, and angiogenesis. Therefore, physical energy can be used as an “energy switch” to control the bone tissue regeneration process or the rate of degradation of the scaffold by turning them on or off.

In addition, we should encourage the use of large-animal bone defect models in future studies. The important role of these models in preclinical research is reflected by the fact that these models can provide a local microenvironment closer to that of human bone defects. Large animals can bridge the gap between small-animal research and clinical translational applications. The innovative development of bone biomaterials is expected to bring new hope to patients with bone nonunion or bone defects, who need bone biomaterials.
